# A 12‐Week Strength Training Improves Mitochondrial Respiration, H_2_O_2_ Emission and Skeletal Muscle Integrity in Women With Myotonic Dystrophy Type 1

**DOI:** 10.1111/apha.70135

**Published:** 2025-11-16

**Authors:** Vincent Marcangeli, Laura Girard‐Côté, Valeria Di Leo, Marie‐Pier Roussel, Conor Lawless, Olivier Charest, Anteneh Argaw, Maude Dulac, Guy Hajj‐Boutros, José A. Morais, Amy Vincent, Gilles Gouspillou, Jean‐Philippe Leduc‐Gaudet, Elise Duchesne

**Affiliations:** ^1^ Department of Physical Activity Sciences Université du Québec à Montréal Montreal Quebec Canada; ^2^ Research Group for Adapted Physical Activity Université du Québec à Montréal Montréal Quebec Canada; ^3^ Department of Biological Sciences Université du Québec à Montréal Montreal Quebec Canada; ^4^ School of Rehabilitation Sciences, Faculty of Medicine Université Laval Quebec City Quebec Canada; ^5^ CHU de Québec—Université Laval Research Center Quebec City Quebec Canada; ^6^ Neuromuscular Diseases Interdisciplinary Research Group (GRIMN) Saguenay‐Lac‐St‐Jean Integrated University Health and Social Services Center Saguenay Quebec Canada; ^7^ Mitochondrial Research Group, Translational and Clinical Research Institute, Faculty of Medical Sciences Newcastle University Newcastle upon Tyne UK; ^8^ NIHR Newcastle Biomedical Research Centre Newcastle upon Tyne Hospitals NHS Foundation Trust and Newcastle University Newcastle upon Tyne UK; ^9^ Department of Fundamental Sciences Université du Québec à Chicoutimi Chicoutimi Québec Canada; ^10^ Research Institute of the McGill University Health Center Montreal Québec Canada; ^11^ John Walton Muscular Dystrophy Research Centre, Clinical and Translational Research Institute, Faculty of Medical Sciences Newcastle University Newcastle UK; ^12^ Research Group in Cellular Signaling, Department of Medical Biology Université du Québec À Trois‐Rivières Trois‐Rivières Quebec Canada; ^13^ Center for Interdisciplinary Research in Rehabilitation and Social Integration (Cirris) Capitale‐National Integrated University Health and Social Services Center Quebec City Quebec Canada

**Keywords:** exercise, mitochondria, mitochondrial function, mitochondrial respiration, myotonic dystrophy, neuromuscular disease, oxidative phosphorylation defects, resistance training, ROS

## Abstract

**Background:**

Myotonic dystrophy type 1 (DM1) is caused by expanded CTG repeats in the *DMPK* gene, causing the accumulation of toxic RNA that sequesters RNA‐binding proteins. Clinically, DM1 is characterized by progressive muscle weakness and atrophy, resulting in reduced physical capacity and quality of life. Recent evidence implicates mitochondrial dysfunction in DM1 pathophysiology. While aerobic exercise has been shown to improve skeletal muscle and mitochondrial health in individuals with DM1, the benefits of strength training remain unexplored.

**Objectives:**

We investigated the effects of a 12‐week strength training program on mitochondrial respiration, reactive oxygen species (ROS) production and muscle integrity in women with DM1.

**Methods:**

Vastus lateralis muscle biopsies were collected pre‐ and post‐training in participants with DM1 and once in unaffected/untrained individuals. Mitochondrial respiration and hydrogen peroxide emission (marker of ROS production) were assessed in permeabilized myofibers, while OXPHOS protein contents were quantified by immunoblotting and immunofluorescence. Markers of myofiber denervation (NCAM+) and integrity (centrally located myonuclei, damaged laminin, nuclear clumps) were assessed on histological sections.

**Results:**

At baseline, DM1 participants exhibited lower mitochondrial respiration compared to unaffected individuals. Strength training significantly improved mitochondrial respiration and content in DM1 participants. At baseline, absolute ROS production was lower, while ROS production normalized to oxygen consumption (free radical leak) was higher, in DM1. Histological signs of denervation and altered muscle integrity were observed. Strength training partially normalized mitochondrial free radical leak and restored some markers of myofiber integrity.

**Conclusion:**

Collectively, our results indicate that strength training enhances mitochondrial health and improves myofiber integrity in women with DM1.

**Summary**

- DM1 is characterized by progressive muscle weakness, atrophy, fatigue, and other symptoms, severely impacting quality of life.
- In addition to toxic RNA accumulation and RNA‐binding protein sequestration, mitochondrial dysfunction is increasingly recognized as a key contributor to DM1 pathogenesis.
- While aerobic exercise has shown benefits, the impact of strength training on mitochondrial health and muscle integrity in women with DM1 remained unexplored.
- Here, we report that a supervised 12‐week strength training program improves mitochondrial respiration and content, reduces mitochondrial ROS, and enhances myofiber integrity in women with DM1.
- These findings position strength training as a safe and effective therapeutic intervention for improving mitochondrial and muscle health in individuals with DM1.

## 1 Introduction

Myotonic dystrophy type 1 (DM1) is an inherited autosomal dominant disease with an estimated prevalence of 1 in 5500 people worldwide [1]. The highest prevalence is found in the Saguenay–Lac‐Saint‐Jean region of Quebec, Canada, where it affects 1 in 633 individuals [2]. DM1 is caused by an abnormal and unstable CTG nucleotide repeat in the 3′‐untranslated region of the *dystrophia myotonica protein kinase* (*DMPK*) gene, resulting in mutant mRNA foci composed of auto‐complementary hairpin structures, which bind and sequester RNA‐binding proteins from the muscleblind‐like (MBNL) family and abnormally stabilize the CUGBP Elav‐like family member 1 (CELF1) [3]. MBNL and CELF1 are involved in numerous cellular functions, including RNA splicing, polyadenylation stability and translation [3]. The disease severity is positively correlated with repeat expansion size and is inversely related to the age of onset [4]. While there are tremendous advances in the understanding of the pathophysiological implications, there is currently no cure for DM1 [5].

DM1 is a heterogeneous disease, with the presence and severity of the symptoms varying significantly among individuals. People with DM1 experience muscular symptoms such as myotonia and muscle atrophy [6], as well as fatigue, daytime sleepiness, and apathy, which significantly affect their quality of life [7, 8, 9]. Muscle weakness and muscle atrophy are cardinal symptoms that are observed in the muscles of the hands, neck, and face, and are also reported in the upper and lower limbs [6, 10, 11]. Muscle weakness restricts mobility and limits participation in social activities [10, 11, 12, 13, 14, 15]. Studies using DM1 mouse models, and human DM1 muscle biopsies have also revealed markers of impaired neuromuscular junction integrity with evidence of muscle fiber denervation in DM1 [16, 17, 18, 19]. Moreover, studies highlighted a predominance and atrophy of type I myofibers [6, 20], as well as higher proportions of fibers with central nuclei, nuclear aggregations and sarcolemma damage [21]. While mRNA toxicity is considered the main pathophysiological mechanism linking the nucleotide expansion to the clinical manifestation of the disease [22], the contribution of other cellular mechanisms is still understudied.

There is a growing body of literature suggesting that mitochondrial dysfunction may play a central role in the pathophysiology of DM1. A study from the early 1990s has reported mitochondrial abnormalities in skeletal muscle of individuals with myotonic dystrophy, such as increased mtDNA deletions [23]. Otherwise, primary cell culture experiments using fibroblasts and peripheral blood mononuclear cells (PBMCs) derived from individuals with DM1 have reported impairments in mitochondrial dynamics and function [24]. In addition, several studies using magnetic resonance spectroscopy have shown altered bioenergetics in both the brain and skeletal muscle of individuals affected by myotonic dystrophy [25, 26]. Interestingly, alterations in mitochondrial oxidative phosphorylation (OXPHOS) likely contribute to clinical manifestations seen in DM1 [26]. Specifically, proteins forming Complexes I and IV are downregulated, potentially causing a reduction in OXPHOS complexes, a decline in ATP level, and an increase in reactive oxygen species (ROS) production [27, 28]. Taken together, these findings suggest that dysregulated mitochondrial content and impaired respiratory capacity are central features of DM1 pathogenesis.

Mitochondria are organelles highly affected by physical activity, and physical activity is considered one of the best ways to improve both mitochondrial and general health [29, 30]. Aerobic and strength training are considered safe for the DM1 population [31]. A 12‐week aerobic exercise program improved cardiorespiratory fitness, muscular endurance, and overall mitochondrial health in muscle biopsies from men and women with DM1 [32]. On the other hand, a 12‐week strength training program in men with DM1 resulted in improvements in maximal knee extensor muscle strength, walking speed, and standing‐up capacity, along with increased myofiber size in a subgroup of participants [33]. Interestingly, the same exercise program showed that strength training partially rescued markers of mitochondrial dysfunction, as it increased Complexes I and IV proteins, in men with DM1 [27], like what was previously reported following an aerobic exercise program [32]. A similar study with an identical strength training regimen was recently conducted with a female cohort. This training program improved muscle strength, apathy, depression, pain interference, and myofiber size [34]. Despite differences in clinical presentation, both males and females with DM1 benefit from strength training, which enhances muscle force and myofiber size. However, whether strength training improves mitochondrial content, respiration, ROS production and markers of muscle fiber integrity in individuals with DM1 remains unknown. Although women make up nearly half of the global population, they remain significantly underrepresented in exercise science research [35]. Furthermore, sex‐specific differences in DM1 clinical presentation underscore the importance of targeted investigations in women. To address these knowledge gaps, the present study was designed to assess the effects of a 12‐week strength training program on markers of mitochondrial content, mitochondrial respiration, ROS production, and various histological markers of muscle fiber integrity in women diagnosed with DM1.

## 2 Methods

### 2.1 Ethical Approval

Each participant provided written informed consent after receiving verbal and written explanations of all procedures and associated risks. All women participants with DM1, as well as four women without DM1 (thereafter called unaffected individuals), were recruited at the *Centre intégré universitaire de santé et de services sociaux* (CIUSSS) du Saguenay‐Lac‐Saint‐Jean, Quebec, Canada, as described in [34]. This study was approved by the Ethics Review Board of the CIUSSS of Saguenay‐Lac‐Saint‐Jean (certificate number: 2019‐063). In addition, data from five women without DM1 (unaffected individuals), who participated in a previous study conducted at the Research Institute of the McGill University Health Centre, were included in the unaffected group [36]. This study was approved and monitored by the McGill University Health Centre Human Research Ethics Board (code: 2021‐7170). Both studies conformed to the ethical standards outlined in the version of the *Declaration of Helsinki* applicable at the time of the experiments.

### 2.2 Study Design and Participants

The inclusion criteria have been described in [34]. Briefly, for the DM1 group, all women participants had a genetically confirmed diagnosis of DM1, were between the ages of 20 and 60, and received approval from their neurologist before starting the intervention. Only DM1 women for whom pre‐ and post‐training muscle biopsies were available were included in this study (ClinicalTrials.govID: NCT05400629). For each participant with DM1, the number of CTG repeats was determined by polymerase chain reaction using DNA isolated from blood samples. Phenotypes were extracted from medical records and anthropometric measurements were collected according to standard procedures. The unaffected participants were required to be women aged between 20 and 65. The unaffected participants from the CIUSSS were recruited through word of mouth, while those from the RI‐MUHC took part in a clinical trial (ClinicalTrials.govID: NCT04964999) [36].

### 2.3 Strength Training Program

Women with DM1 underwent a 12‐week lower limb strength training program as previously described [34]. Briefly, they followed a training regimen twice a week for 12 weeks under the supervision of a physiotherapist or kinesiologist. They performed three sets of six to eight maximum repetitions with progressive loading in the following exercises: squat, leg press, knee extension, hip abduction, and plantar flexion. A rest period of 2 min was observed between sets.

### 2.4 Skeletal Muscle Biopsies

Muscle biopsies were collected from the *vastus lateralis* muscle from every participant with DM1 within 1 week before and after the 12‐week training program and only once from unaffected participants. All biopsies were performed in the morning, after an overnight fasting period. The biopsies were obtained using a Bergström muscle needle, through an incision made 15 cm above the patella, under local anesthesia with 2% xylocaine. Each muscle biopsy was divided into multiple portions and stored accordingly for subsequent biochemical and histological experiments. More precisely, a portion of fresh muscle tissue was used to assess mitochondrial function (approximately 50 mg); another portion was mounted in tragacanth (Sigma–Aldrich #G1128) on plastic blocks and frozen in pre‐cooled isopentane in liquid nitrogen; and the remaining pieces were flash‐frozen in liquid nitrogen for biochemical analyses. All frozen samples were stored at −80°C until further use.

### 2.5 Preparation of Permeabilized Muscle Fibers

Mitochondrial function was assessed using fresh muscle biopsy samples. Once dissected, muscle biopsy samples were weighted with a precision scale and then rapidly immersed in ice‐cold stabilizing buffer A (2.77 mM CaK_2_ ethylene glycol‐bis‐(2‐aminoethylether)‐N,N,N,N‐tetraacetic acid (EGTA), 7.23 mM K_2_ EGTA, 6.56 mM MgCl_2_, 0.5 mM dithiothreitol (DTT), 50 mM 2‐(N‐morpholino) ethanesulfonic acid potassium salt (K‐MES), 20 mM imidazol, 20 mM taurine, 5.3 mM Na_2_ ATP, and 15 mM phosphocreatine, pH 7.3). Muscle biopsy samples were separated into small fiber bundles using fine forceps under a surgical dissecting microscope (Leica S4 E, Germany). Muscle fiber bundles were incubated into a glass scintillation vial for 30 min at low rocking speed containing buffer A supplemented with 0.05 mg/mL saponin (Sigma‐Aldrich #S7900) to selectively permeabilize the sarcolemma. Fiber bundles were then washed three times for 10 min each at a low rocking speed in MiR05 buffer (110 mM sucrose, 20 mM HEPES, 10 mM KH_2_PO_4_, 20 mM taurine, 60 mM K‐lactobionate, 3 mM MgCl_2_, 0.5 mM EGTA, 1 g/L of fatty acid free BSA, pH 7.4) to assess mitochondrial bioenergetics and H_2_O_2_ emission. The muscle biopsies from RI‐MUHC were processed and analyzed under the same conditions as those from the CIUSSS, using the same equipment and solutions.

### 2.6 Assessment of Mitochondrial Respiration

Mitochondrial respiration was assessed on permeabilized muscle fibers at 37°C in 2 mL of MiR05 buffer in an Oroboros O2K high‐resolution fluororespirometer (Oroboros Instruments, Austria). Briefly, 3 to 6 mg (wet mass) of permeabilized fiber bundles were weighed and added to the respiration chambers. The following substances were used: glutamate (10 mM) and malate (5 mM), ADP (2 mM), succinate (10 mM), oligomycin (1 μM) and antimycin A (2 μM). Respiration rates were normalized as nanomoles of dioxygen per minute per mg of wet muscle mass (nmolO_2_ min^−1^ mg^−1^). All respiration experiments were analyzed with MitoFun (https://zenodo.org/records/7510439), a homemade code in the Igor Pro 8 software (Wavemetrics, OR, USA).

### 2.7 Assessment of Mitochondrial H_2_O_2_ Emission

The myofiber bundles peroxide emission was assessed by monitoring the rate of H_2_O_2_ release using the Amplex Ultra Red‐horseradish peroxidase [37] system. This was performed along with respiration assessment at 37°C in 2 mL of MiR05 buffer supplemented with Amplex Ultra Red (10 μM), SOD (5 U/mL), and HRP (1 U/mL) in the Oroboros O2K high‐resolution fluororespirometer (Oroboros Instruments, Austria). A calibration curve was generated daily using successive additions of known [H_2_O_2_] in the absence of tissues. H_2_O_2_ emission was normalized as picomoles per minute per milligram of wet muscle mass (pmolH_2_O_2_.min^−1^ mg^−1^). All H_2_O_2_ emission experiments were analyzed with MitoFun (https://zenodo.org/records/7510439), a homemade code to analyze mitochondrial function data in the Igor Pro 8 software (Wavemetrics, OR, USA).

### 2.8 Immunoblotting

Portions of frozen muscle tissues were homogenized in ice‐cold lysis buffer (50 mM HEPES, 150 mM NaCl, 100 mM NaF, 5 mM EDTA, 0.5% Triton X‐100, 0.1 mM DTT, 2 μg/mL leupeptin, 100 μg/mL PMSF, 2 μg/mL aprotinin, and 1 mg/100 mL pepstatin A, pH 7.2) with ceramic beads using Mini‐Beadbeater (BioSpec Products, Bartlesville, OK) at 60 Hz. Muscle homogenates were kept on ice for 30 min with periodic agitation and then centrifuged at 14000 × *g* for 15 min at 4°C; supernatants were collected, and pellets were discarded. The protein content in each sample was determined using the Bradford or BCA (Pierce) method. Aliquots of crude muscle homogenate were mixed with Laemmli buffer (6×, reducing buffer, Boston BioProducts #BP111R) and subsequently denatured for 5 min at 95°C. Equal amounts of protein extracts (15 μg per lane) were separated on 4%–15% gradient stain‐free gels (Mini‐PROTEAN TGX Stain‐Free Gels, Bio‐Rad Laboratories) by SDS‐PAGE and subsequently transferred to polyvinylidene difluoride (PVDF) (Bio‐Rad Laboratories). The total proteins on membranes were detected with stain‐free technology from Bio‐Rad. Membranes were blocked in PBS + 1% Tween 20 + 5% bovine serum albumin (BSA) or TBS + 1% Tween 20 + 5% milk for 1 h at room temperature and then incubated overnight at 4°C with Total OXPHOS antibody cocktail (Abcam #ab110413) composed of monoclonal antibodies targeting the NADH dehydrogenase beta subcomplex subunit 8 (NDUFB8), succinate dehydrogenase subunit B (SDHB), cytochrome b‐c1 complex subunit 2 (UQCRC2), cytochrome c oxidase subunit 1 (MT‐CO1), and ATP synthase subunit alpha (ATP5A). Bands were revealed using the ECL‐Plus chemiluminescent detection system (Bio‐Rad, Clarity ECL substrate, 170‐5060) and imaged with the ChemiDoc Imaging System. The optical densities (OD) of protein bands were quantified using ImageLab 6.1 software (Bio‐Rad Laboratories) and normalized to the total protein signal obtained from the stain‐free blot image of the corresponding sample, which served as the loading control. Immunoblotting data are expressed as relative to unaffected participants.

### 2.9 Histological Analyses

#### 2.9.1 Skeletal Muscle Sample Sectioning

Muscle biopsy samples were cryosectioned at a thickness of 10 μm using a cryostat maintained at −20°C, then mounted onto Superfrost slides for subsequent histological staining analyses.

#### 2.9.2 Quadruple Immunofluorescence

Muscle slices from nine DM1 and four unaffected participants were sent in dry ice to the Mitochondrial Research Group at Newcastle University, Newcastle, United Kingdom to perform a quadruple immunofluorescence (QIF) analysis. Muscle cryosections were labeled by the QIF technique, as previously described [38]. NDUFB8 and MT‐CO1, which are OXPHOS subunits of Complexes I and IV, respectively, the voltage‐dependent anion channel (VDAC1) as a mitochondrial mass marker and laminin as a cell membrane marker were detected using antibodies (Table S1). Negative primary controls labeled only with anti‐laminin were processed in parallel to each of the samples included in the QIF. Fluorescent images were taken with Zeiss Axioscan 7 and analyzed using Zen 2011 (blue edition) software. The Zeiss Axioscan 7 was set to use an Axiocam 712 mono camera, a 10×/0.45 M27 objective lens, and an X‐cite Xylis LED module as the light source. Image acquisition was performed at 20× magnification using a motorized automated stage and the tiling function in Zen software. For each section, a .czi file was generated using the stitching function in Zen. Images of the skeletal muscle sections were processed as previously described [27]. Laminin signal was used to create a segmentation map for each of the sections with *Quadruple Immuno Analyzer* [38]. An unaffected population of skeletal muscle fibers was used to build a 95% predictive interval linear regression model. Fibers from participants with DM1's samples before and after strength training resting within the unaffected predictive interval were classified as normal for NDUFB8 and MT‐CO1, whereas those lying below or above this interval were classified as fibers with low (Δfibers^low^) or high level (Δfibers^high^), respectively. Changes in the proportion of fibers with a high level of complexes I and IV were classified as significant when there was an increase after intervention (Δfibers^high^ > 0) and *p* < 0.05. Similarly, changes in the proportion of fibers with a low level of Complex I and Complex IV were classified as significant when there was a decrease after intervention (Δfibers^low^ < 0) and *p* < 0.05.

#### 2.9.3 Assessment of Muscle Fiber Integrity

NCAM‐dystrophin staining was used as a marker to assess neuromuscular junction integrity, specifically as an indicator of denervation. First, muscle cross‐sections were allowed to reach room temperature before being incubated for 15 min in a permeabilization solution (0.1% Triton X‐100 in PBS). Slides were then washed three times with PBS and blocked with goat serum (10% in PBS) for 1 h. After that, muscle cross‐sections were incubated for 2 h at room temperature with mouse monoclonal IgG1 anti‐NCAM (BD Biosciences, #347740; 1:50 dilution) and mouse monoclonal IgG2b anti‐dystrophin (Milipore Sigma #D8168; 1:500 dilution) (Table S1). Sections were then washed three times in PBS before being incubated for 1 h at room temperature with Alexa Fluor 594 goat anti‐mouse IgG1 (Thermo‐Fischer Scientific, #A‐21125, 1:500) and Alexa Fluor 488 goat anti‐mouse IgG2b (Thermo‐Fischer Scientific, #A‐21141, 1:500) antibodies. Sections were then washed again three times for 10 min with PBS and finally cover slipped using Prolong Diamond (ThermoFisher #P36961) as mounting medium. Slides were imaged with a Zeiss fluorescence microscope (Zeiss Axio Imager 2). The proportion of NCAM‐positive fibers was manually quantified using ImageJ.

#### 2.9.4 Measurement of Central Nuclei, Nuclear Aggregations, and Laminin Damage

Laminin‐DAPI staining was used to assess central nuclei, nuclear aggregations, and laminin damage in muscle biopsy cross‐sections (Table S1). Cross‐sections were brought to room temperature, rehydrated with PBS, and subsequently blocked with goat serum (10% in PBS) for 30 min. Following blocking, sections were incubated for 1 h at room temperature with a primary polyclonal anti‐laminin rabbit IgG antibody (Sigma‐Aldrich, #L9393; 1:750 dilution). Sections were then washed three times in PBS before being incubated for 1 h at room temperature with an Alexa Fluor 594 goat anti‐rabbit IgG antibody (Thermo‐Fischer Scientific, #A‐11037, 1:500). Sections were then washed three times in PBS and slides were cover slipped using Prolong Gold with Dapi (Invitrogen, #P36931) as a mounting medium. Slides were imaged using a Zeiss fluorescence microscope (Zeiss Axio Imager). Each muscle fiber within the section was evaluated for the presence of at least one central nucleus, an aggregation of two or more nuclei, and damage to the laminin membrane. Each slide was analyzed with ImageJ2 software (version 2.9). For each variable, a minimum of 100 myofibers was analyzed per sample. Missing values were due to technical errors and/or insufficient remaining tissue availability.

### 2.10 Statistical Analysis

Statistical tests were performed in GraphPad Prism 10.4.1 (GraphPad Software, Boston, MA, USA). The acceptor control ratio (ACR), an indicator of mitochondrial coupling efficiency, was determined by dividing state III (glutamate + malate + ADP) by state II (glutamate + malate) rates of respiration. OXPHOS protein content index for DM1 participants was normalized by the mean of the unaffected participants. Mann–Whitney tests were used to investigate any significant difference in the means of independent populations (unaffected vs. DM1‐PRE and unaffected vs. DM1‐POST) and Wilcoxon matched‐pairs signed rank tests were used to compare DM1‐PRE vs. DM1‐POST and DM1‐PRE NCAM+ and NCAM− cross‐sectional area. Two‐way ANOVAs with Šídák's post hoc test to adjust *p*‐values for multiple comparisons were used to assess differences between unaffected individuals and DM1‐PRE intervention and between unaffected individuals and DM1‐POST intervention. Repeated measures two‐way ANOVA with Šídák's post hoc test to adjust *p*‐values for multiple comparisons was conducted to compare DM1‐PRE vs. DM1‐POST intervention. For QIF analysis, mean values were used for comparisons with bilateral *t*‐tests adjusted with the Bonferroni method. Also, a combined method with bootstrapping, permutation and false discovery rate testing was used to assess statistical uncertainty and significant changes [27]. Detailed information on statistical testing is provided in each figure legend. *p*‐values < 0.05 were considered statistically significant and *p*‐values < 0.1 were reported as trends.

## 3 Results

### 3.1 Baseline Participant Characteristics

Nine women with juvenile, adult or late DM1 phenotypes, aged between 24 and 52 years (mean age: 36 years) were included in the present study along with nine age‐matched unaffected women. Detailed participant characteristics are presented in Table 1. At baseline, there was no difference in age, weight, height and body mass index (BMI) between the unaffected individuals and DM1 participants (Table 1).

**TABLE 1:** Participants' characteristics at baseline.

Participant number	DM1 phenotype	Age (years)	Weight (kg)	Height (cm)	BMI (kg m^−2^)	CTG (*n*)	NCAM+ fibers (%)
Unaffected individuals							
Unaffected_1	n/a	24	54.0	165	19.8	n/a	0.35
Unaffected_2	n/a	32	68.0	170	23.5	n/a	0.31
Unaffected_3	n/a	22	61.2	159	24.2	n/a	No data
Unaffected_4	n/a	25	70.3	165	25.8	n/a	2.40
Unaffected_5	n/a	59	67.2	159	26.7	n/a	0.94
Unaffected_6	n/a	58	52.2	157	21.3	n/a	0.75
Unaffected_7	n/a	57	58.8	165	21.5	n/a	0.88
Unaffected_8	n/a	63	74.1	159	29.3	n/a	0
Unaffected_9	n/a	60	56.1	162	21.4	n/a	No data
Mean (SD)	n/a	44 ± 18	62.4 ± 7.8	162 ± 4	23.7 ± 3.1	n/a	0.80 ± 0.78
DM1 participants							
P1	Juvenile	52	56.0	150	24.9	509	7.14
P2	Juvenile	33	52.5	158	21.0	462	0
P3	Juvenile	33	40.0	168	14.2	832	61.35
P4	Late	39	80.2	167	28.8	70	0.69
P5	Adult	52	42.8	165	15.7	466	8.70
P6	Juvenile	24	47.6	162	18.1	488	6.96
P7	Adult	37	78.5	153	33.5	432	2.07
P8	Juvenile	25	80.0	161	30.9	300	1.15
P9	Adult	30	84.3	161	32.5	324	1.39
Mean (SD)	n/a	36 ± 10	62.4 ± 18.1	161 ± 6.1	24.4 ± 7.4	431 ± 204	9.94 ± 19.55
*p*	n/a	0.422	0.931	0.714	0.931	n/a	0.057

*Note:* Mann–Whitney tests were used to compare the means of unaffected and DM1 participants.

Abbreviations: BMI, body mass index; n/a, not applicable; SD, standard deviation.

### 3.2 Strength Training Partially Rescued Mitochondrial Respiration in Women With DM1

**FIGURE 1:**
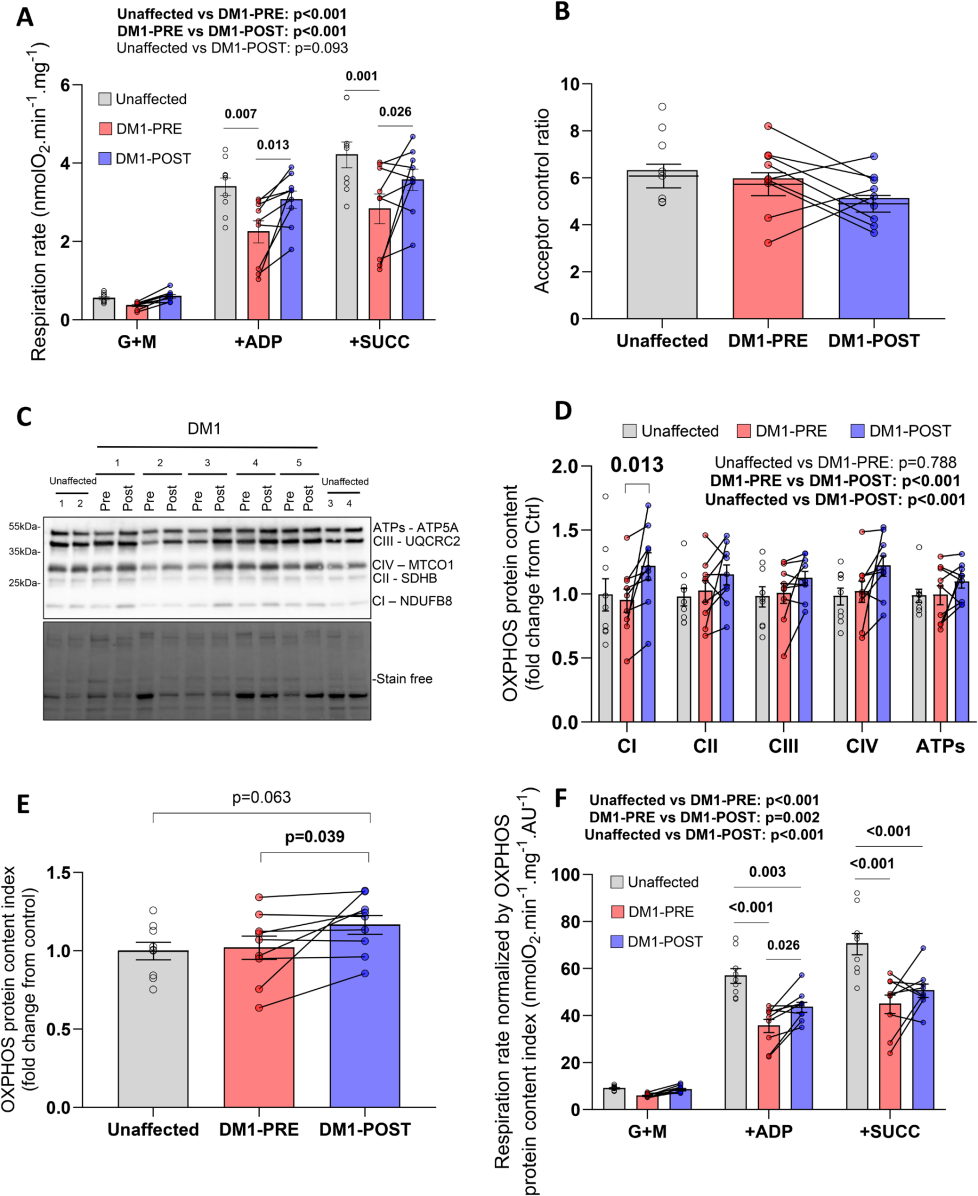
The impacts of a 12‐week supervised strength training program on mitochondrial respiration and content in women diagnosed with DM1. (A) Mitochondrial respiration rates in permeabilized myofibers prepared from *vastus lateralis* biopsy samples obtained from participants in the unaffected and DM1 groups using the following sequential additions of substrates: Glutamate + malate (G + M; Complex I substrates), ADP and succinate (SUCC; Complex II substrate). (B) Acceptor control ratio (ACR) was determined by dividing the respiration rate obtained with glutamate + malate + ADP by the respiration rate obtained when only glutamate + malate were present. (C) Representative immunoblots of subunits of Complex I (CI), Complex II (CII), Complex III (CIII), Complex IV (CIV) and ATP synthase (ATPs) performed on *vastus lateralis* muscle samples obtained from unaffected and DM1 participants. (D) Quantification of OXPHOS subunits content expressed as fold change relative to the unaffected group. (E) OXPHOS protein content index calculated as the sum of OXPHOS representative subunits. (F) Mitochondrial respiration rates normalized by OXPHOS protein content index in the unaffected and DM1 groups using the following sequential additions of substrates: Glutamate + malate (G + M; Complex I substrates), ADP and succinate (SUCC; Complex II substrate). Each mitochondrial respiration experiment was conducted in duplicate (unaffected group: *n* = 9; DM1 pre: *n* = 9; DM1 post: *N* = 9). Two two‐way ANOVAs with Šídák's post hoc test to adjust *p*‐values for multiple comparisons were used to assess differences between unaffected and DM1‐PRE and unaffected and DM1‐POST for graphs A, D and F. Repeated measures two‐way ANOVA with Šídák's post hoc test to adjust *p*‐values for multiple comparisons were used to assess differences between DM1‐PRE and DM1‐POST for graphs A, D, and F. Mann–Whitney tests were used to compare unaffected and DM1 groups for graphs B and E. Wilcoxon matched‐pairs signed rank tests were used to compare DM1‐PRE and DM1‐POST for graphs B and E. *p* < 0.05 was considered statistically significant and *p* < 0.1 was reported as a trend. All data in bar graphs are presented as means ± SEM.

Mitochondrial respiration was assessed with high‐resolution fluororespirometry on permeabilized myofibers isolated from biopsies of the *vastus lateralis* muscle to determine whether strength training improves mitochondrial respiration in women participants with DM1. At baseline, DM1 participants displayed lower mitochondrial respiration rates compared to unaffected individuals, which were partially restored by the 12‐week strength training program (Figure 1A). This effect was particularly pronounced for maximal ADP‐stimulated (state III) respiration rates driven by Complex I (glutamate + malate) and Complex I plus II substrates (glutamate + malate + succinate). The acceptor control ratio (ACR), an indirect indicator of the coupling efficiency between mitochondrial oxygen consumption and ATP production, was also calculated (Figure 1B). There was no difference in the ACR between the two groups, suggesting that the mitochondrial coupling efficiency remained unaffected by DM1 and/or strength training. Taken altogether, these results show that a 12‐week strength training program is effective in improving mitochondrial respiration in women with DM1.

To assess if the observed improvements in mitochondrial respiration rates were attributable to an increase in the abundance of the OXPHOS complexes, we evaluated the levels of representative OXPHOS subunits via immunoblotting (Figure 1C). Before the intervention, DM1 participants exhibited OXPHOS subunit levels comparable to those of unaffected individuals (Figure 1D). A significant group effect was observed when comparing DM1‐PRE and DM1‐POST intervention, indicating an overall increase in OXPHOS subunit content in response to 12 weeks of strength training in DM1 participants. Multiple comparisons indicated that this increase was particularly pronounced for the representative subunit of Complex I (Figure 1D). An OXPHOS protein content index was then calculated, revealing that the sum of all the subunits was similar between the DM1‐PRE and unaffected groups (Figure 1E). Strength training increased the OXPHOS protein content index in DM1 participants (*p* = 0.039) and there was a trend for a higher OXPHOS protein content index between unaffected and DM1‐POST intervention groups (Figure 1E, *p* = 0.063). To assess normalized mitochondrial respiration relative to mitochondrial content, we then divided the mitochondrial respiration rates by the OXPHOS protein content index (Figure 1F). When compared to unaffected individuals, DM1 participants showed significantly lower rates of normalized maximal ADP‐stimulated (state III) respiration rates driven by complexes I and II substrates (glutamate + malate + succinate) (Figure 1F). Strength training improved normalized mitochondrial respiration, an effect particularly noticeable for state III (ADP‐stimulated) respiration (Figure 1F). Normalized respiration was still significantly lower between DM1 post‐intervention in comparison to unaffected individuals (Figure 1F), indicating that the observed improvements represent a partial rescue. Taken altogether, these findings indicate that strength training improved mitochondrial content and respiration efficiency in DM1 participants.

### 3.3 Strength Training Enhanced the Mitochondrial Content of Fibers in OXPHOS Subunits of Complex I and IV

**FIGURE 2:**
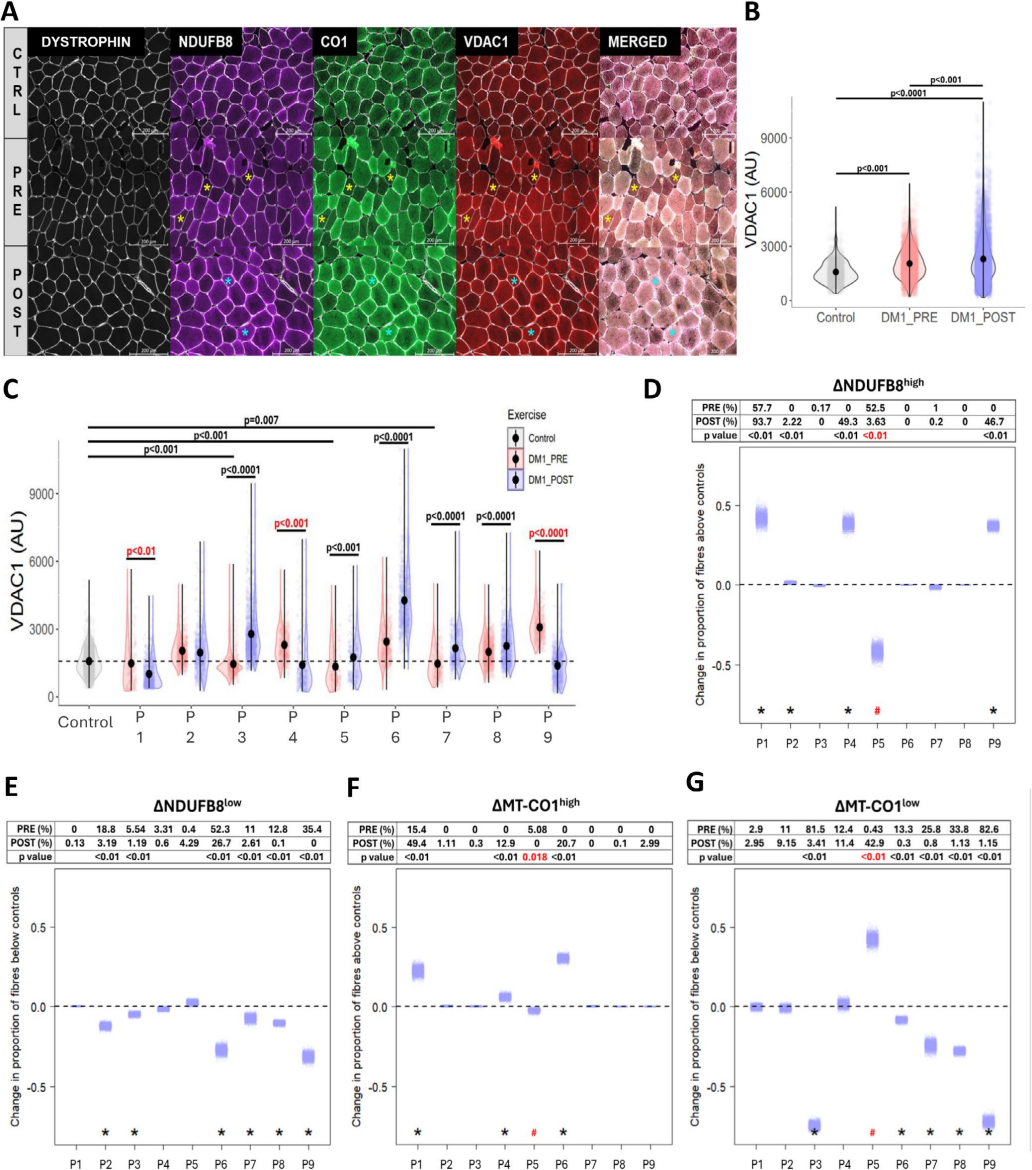
The impacts of a 12‐week supervised strength training program on fibers with low or high Complex I and IV subunits and VDAC1 content in women diagnosed with DM1. (A) Representative images of quadruple immunofluorescence staining in unaffected individuals and DM1 participants. (B) Mitochondrial mass was assessed from participants in the unaffected and DM1 groups with VDAC1. (C) Mean values for unaffected participants (dashed black line) and individual values of each DM1 participant for VDAC1. (D) Delta changes in the proportion of high NDUFB8 fibers in DM1 participants. (E) Delta changes in the proportion of low NDUFB8 fibers in DM1 participants. (F) Delta changes in the proportion of high MT‐CO1 fibers in DM1 participants. (G) Delta changes in the proportion of low MT‐CO1 fibers in DM1 participants. *p* < 0.05 was considered statistically significant. *p*‐values in red and # in red indicate significant negative changes. Yellow stars illustrate deficient fibers (low); cyan stars indicate overabundant fibers (high). For graph B, mean values were used for comparisons with bilateral *t*‐tests adjusted with Bonferroni method, *p*‐value < 0.05 (*). For graphs D, E, F, G changes in the proportion of fibers between post‐ and pre‐exercise cases were calculated and bootstrapped to give estimates of uncertainty and significance of changes were tested using permutation test. Bilateral *t*‐tests between mean values of the proportion of fibers is indicated for each participant with DM1.

To gain a deeper understanding of the changes in mitochondrial content, we performed a QIF staining to analyze modulations occurring within the muscle fibers (Figure 2A). DM1 participants displayed a significantly higher level of VDAC1, an indicator of mitochondrial content, compared to unaffected individuals both before and after intervention (Figure 2B). There was a significant increase in mitochondrial content between pre‐ and post‐exercise in DM1 participants (Figure 2B). At the individual level, we found that five out of nine DM1 participants exhibited increased mitochondrial content after strength training (Figure 2C). However, following the training, three participants (P1, P4, and P9) showed decreased VDAC1 levels while exhibiting a notable increase in the proportion of myofibers having high levels of Complex I subunit (ranging from 47% to 94%; Figure 2D). Additionally, two out of these three participants (P1 and P4) exhibited a higher proportion of myofibers with elevated levels of Complex IV subunit, exceeding those observed in the unaffected group (Figure 2F). Notably, only one participant (P1) from this subgroup exhibited a higher proportion of myofibers with elevated levels of both Complex I and Complex IV subunits, prior to training, compared to the unaffected group (Figure 2D,F). Interestingly, before training, two participants (P1 and P5) expressed a very high proportion of myofibers having elevated levels of Complex I (Figure 2D) and IV (Figure 2F) subunits, with also a very low proportion of myofibers displaying low levels of Complex I (Figure 2E) and IV (Figure 2G) subunits. These two responded very differently to training, with P5 presenting an opposite pattern compared to the rest of the DM1 cohort, where a significant decrease of both Complex I^high^ and Complex IV^high^, and a significant increase of Complex IV^low^ were reported after intervention but still had an improvement in VDAC1.

### 3.4 Strength Training Decreased Mitochondrial H_2_O_2_ Emission in DM1 Participants

**FIGURE 3:**
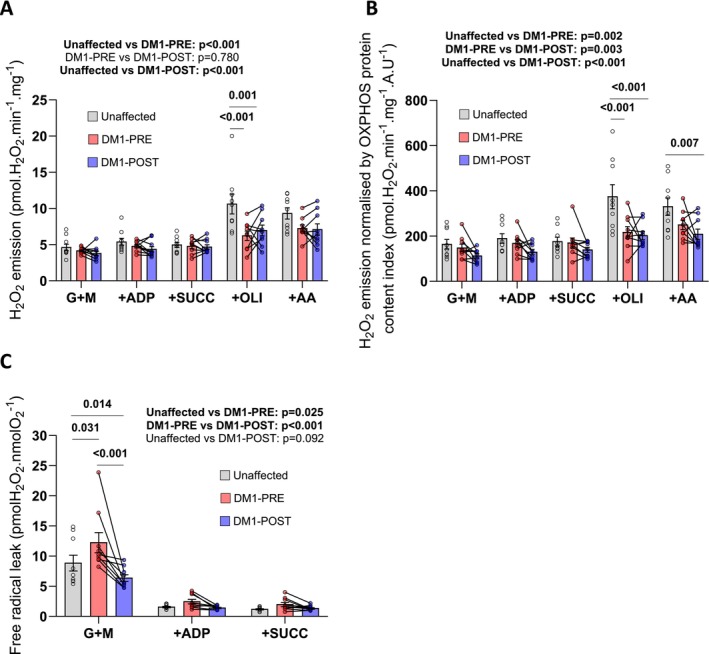
The impacts of a 12‐week supervised strength training program on mitochondrial H_2_O_2_ emission in women diagnosed with DM1. (A) mitochondrial H_2_O_2_ emission rate in permeabilized myofibers prepared from *vastus lateralis* biopsy samples obtained from participants in the unaffected and DM1 groups using the following sequential additions of substrates: Glutamate + malate (G + M), ADP, succinate (SUCC), oligomycin (OLI), antimycin A (AA). (B) Mitochondrial H_2_O_2_ emission normalized by OXPHOS protein content index, driven by glutamate, malate, ADP, succinate, oligomycin and antimycin A in unaffected and DM1 participants. (C) Free radical leak was assessed as the H_2_O_2_ emission rate driven by glutamate + malate + ADP + succinate divided by the corresponding value for mitochondrial respiration. Each mitochondrial H_2_O_2_ experiment was performed in duplicate (unaffected group: *n* = 9; DM1 pre: *n* = 9; DM1 post: *N* = 9). Two two‐way ANOVAs with Šídák's post hoc test to adjust *p*‐values for multiple comparisons were used to assess differences between unaffected and DM1‐PRE, and between unaffected and DM1‐POST intervention. Repeated measures two‐way ANOVA with Šídák's post hoc test to adjust *p*‐values for multiple comparison tests were used to assess differences between DM1‐PRE and DM1‐POST. *p* < 0.05 was considered statistically significant and *p* < 0.1 was reported as a trend. All data in bar graphs are presented as means ± SEM.

Next, we assessed mitochondrial H_2_O_2_ emission as a surrogate for ROS production. As shown in Figure 3A, H_2_O_2_ emission was, overall, lower between DM1 and unaffected participants, which seemed mainly driven by a lower H_2_O_2_ emission rate measured after the addition of oligomycin (an ATP synthase inhibitor) in DM1 participants. There was no difference between DM1‐PRE and DM1‐POST participants regarding absolute mitochondrial H_2_O_2_ emission (Figure 3A). We next normalized H_2_O_2_ emission by the OXPHOS protein content index (Figure 3B) to probe for potential intrinsic changes in H_2_O_2_ emission. As shown in Figure 3B, DM1 participants had an overall lower normalized mitochondrial H_2_O_2_ emission compared to unaffected individuals, which again seemed mainly driven by the H_2_O_2_ emission rate in the presence of oligomycin. An overall group effect was identified when the DM1‐PRE and DM1‐POST were compared, indicating that the 12‐week strength training program resulted in a slight but significant decrease in normalized mitochondrial H_2_O_2_ emission (Figure 3B). As H_2_O_2_ emission was assessed at the same time as respiration, we divided the H_2_O_2_ emission rate by the respiration rate to assess free radical leak (Figure 3C). Interestingly, DM1‐PRE had an overall higher rate of free radical leak than unaffected individuals, and this difference was particularly pronounced under state II conditions (in the absence of ADP). After a 12‐week strength training program, the overall level of free radical leak was significantly reduced in DM1 participants, particularly under state II conditions (Figure 3C). Overall, these data indicate that DM1 participants have lower absolute mitochondrial H_2_O_2_ emission rates but higher free radical leak. Interestingly, 12‐week strength training partially normalized the mitochondrial free radical leak.

### 3.5 Strength Training Positively Impacted Muscle Fiber Integrity in DM1

**FIGURE 4:**
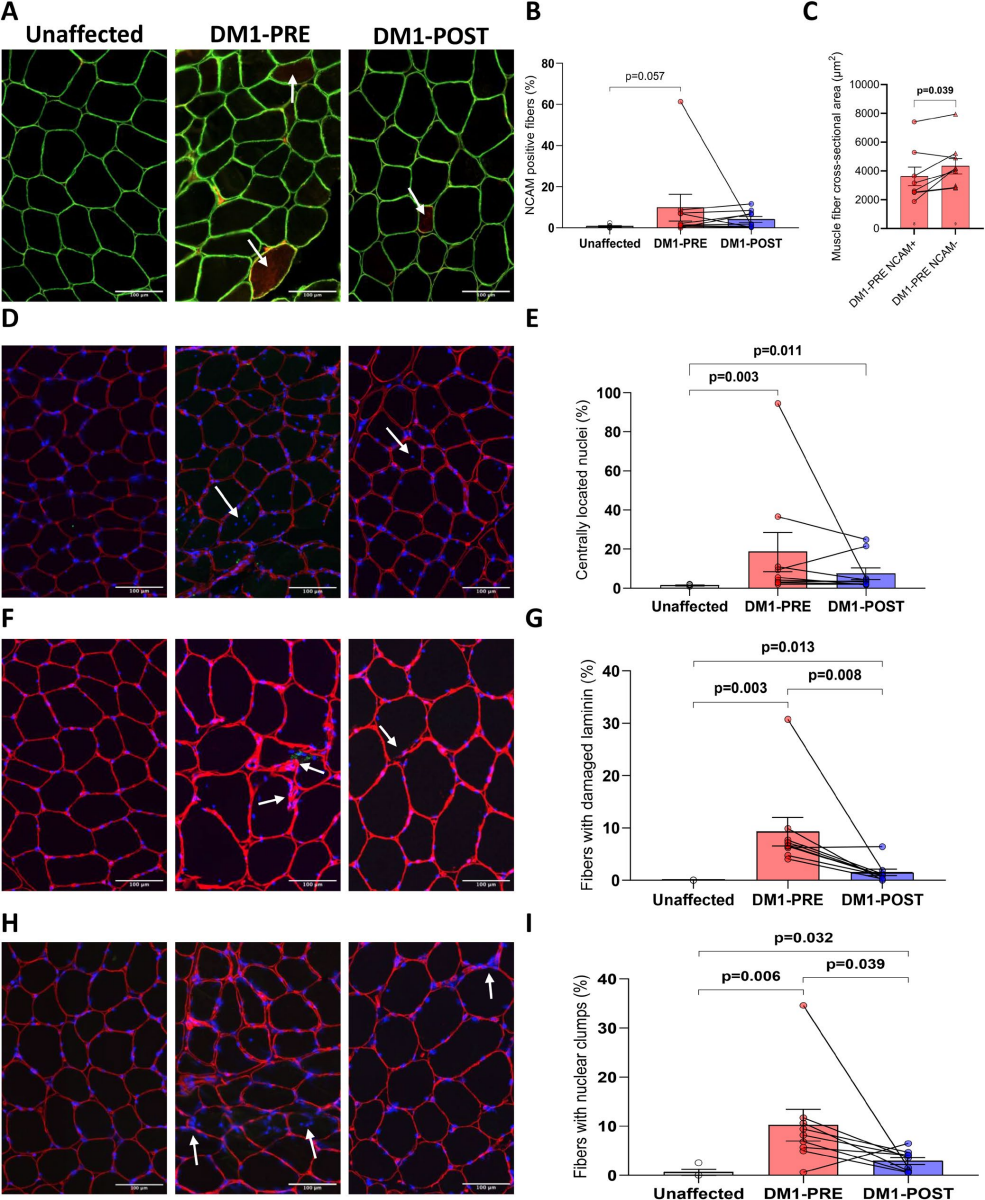
The impacts of a 12‐week supervised strength training program on markers of muscle fiber denervation and integrity in women with DM1. (A) Representative immunolabeling for the denervation marker neural cell adhesion molecule (NCAM; in red) and dystrophin (green) obtained on *vastus lateralis* cross‐sections (scale bar: 100 μm). (B) Quantification of the proportion of NCAM‐positive myofibers (unaffected group *n* = 7 and DM1 groups *n* = 9). (C) Muscle fiber cross‐sectional area (CSA) of NCAM+ and NCAM− fibers in participants with DM1 at baseline. (D) Representative immunolabeling for centrally located nuclei (blue) and laminin (red) (scale bar: 100 μm). (E) Quantification of the proportion of centrally located nuclei (unaffected group *n* = 4 and DM1 groups *n* = 9). (F) Representative immunolabeling for fibers with damaged laminin (red) (scale bar: 100 μm). (G) Quantification of the proportion of fibers with damaged laminin (unaffected group *n* = 4 and DM1 groups *n* = 9). (H) Representative immunolabeling for fibers with nuclear clumps (blue) (scale bar: 100 μm). (I) Quantification of the proportion of fibers with nuclear clumps (unaffected group *n* = 4 and DM1 groups *n* = 9). Mann–Whitney tests were used to compare unaffected and DM1 groups. Wilcoxon matched‐paired‐pairs signed rank tests were used to compare DM1 pre‐ and post‐intervention and DM1‐PRE NCAM+ and NCAM− CSAs. *p* < 0.05 was considered statistically significant and *p* < 0.1 was reported as a trend. All data in bar graphs are presented as means ± SEM.

We assessed muscle fiber denervation using NCAM as a marker [39, 40] (Figure 4A,B). There was a tendency for DM1 participants to have higher proportions of NCAM‐positive fibers compared to unaffected individuals (*p* = 0.057) (Figure 4B). It is worth noting that even if the DM1 participant with the highest NCAM value is removed from this analysis, the trend remains (*p* = 0.097, data not shown). Although there was no significant change in the number of NCAM‐positive fibers after the intervention in the DM1 group, the post‐intervention DM1 group showed comparable levels to unaffected individuals (Figure 4B). At baseline, the cross‐sectional area of NCAM+ fibers was significantly lower (−17.8%) compared to NCAM− fibers, supporting the presence of genuine denervation in DM1 participants (Figure 4C). Next, we evaluated muscle fiber integrity by quantifying the proportion of fibers with central nuclei (Figure 4D,E), damaged laminin (Figure 4F,G), and nuclear clumps (Figure 4H,I). Before the intervention, DM1 participants had higher levels of centrally nucleated fibers (Figure 4E), damaged laminin (Figure 4G) and nuclear clumps (Figure 4I) than unaffected individuals. Strength training significantly decreased the proportion of myofibers with damaged laminin and nuclear clumps (Figure 4G,I). It is worth noting that removing the DM1 participant with the highest values only affects the comparison between DM1‐PRE and DM1‐POST training for the proportion of fibers with nuclear clumps, reducing the significant difference to a trend (*p* = 0.078, data not shown). However, post‐intervention, DM1 participants still showed a significantly higher proportion of fibers with central nuclei, damaged laminin and nuclear clumps than unaffected individuals (Figure 4D–I). Taken altogether, these data indicate that a 12‐week strength training program improves muscle fiber integrity.

## 4 Discussion

DM1 manifests through various symptoms, notably loss of muscle mass and strength, which impact quality of life [6]. Increasing evidence suggests that mitochondrial dysfunction in skeletal muscle plays a critical role in the pathophysiology of DM1 [25, 26, 32]. Given the well‐established benefits of physical activity, notably on mitochondrial health [29, 41], exercise training offers huge therapeutic potential for individuals with DM1. Recently, Mikhail et al. [32] reported that moderate aerobic exercise improved mitochondrial respiration and dynamics in individuals with DM1. To our knowledge, no prior study has examined the impact of strength training on mitochondrial content and bioenergetics in individuals with DM1. Despite growing awareness of the underrepresentation of women's data in research and in exercise science, efforts to address this disparity are ongoing and take time to resolve [35, 42, 43]. Notably, DM1 presents sex‐specific differences in clinical presentation [44], as men with DM1 experience more severe muscular impairments, whereas women tend to show milder muscular symptoms, but more systematic clinical manifestations, supporting the need for sex‐targeted investigations. Therefore, this study focused exclusively on women participants with DM1. We report that a 12‐week strength training program leads to significant improvements in skeletal muscle health in women with DM1, including increased mitochondrial content, better ADP‐stimulated mitochondrial respiration, and lowered normalized H_2_O_2_ emission and free radical leak. Additionally, there was a significant reduction in the proportion of skeletal muscle fibers with damaged laminin and nuclear clumps in women with DM1.

Our results show significant alterations in mitochondrial respiration in women with DM1 compared to unaffected individuals. This low mitochondrial respiration in DM1 aligns with previous findings [27, 32]. We report that strength training improved mitochondrial respiration, with effects comparable to those seen after aerobic training [32]. These findings reinforce our recent study demonstrating that strength training mitigates mitochondrial dysfunction in skeletal muscles of men with DM1 [27]. Similar training‐induced improvements have also been observed in the DM1 mouse model [45, 46]. Individuals with DM1 have generally lower physical activity levels than healthy individuals, a difference that is largely attributed to disease‐related functional limitations [47]. Additionally, some healthcare professionals do not recommend exercise to individuals with DM1 as they continue to believe that those with neuromuscular diseases should avoid physical activity [48]. This overly cautious approach stems from concerns that exercise could potentially worsen the condition [48]. However, it is safe for this population to do both strength and aerobic training [31]. Our team also conducted two 12‐week strength training interventions in both men and women affected by DM1, and no major negative side effects were reported on top of having improvement in lower limb strength and function [33, 34]. We suggest that even small increases in physical activity could positively impact mitochondrial respiration in individuals with DM1. Interestingly, our results showed that before the intervention, markers of mitochondrial content were comparable (OXPHOS protein content) or even elevated (VDAC1) in DM1 participants. Previous studies have reported splicing defects in mitochondrial genes such as OPA1 and DNM1L in DM1 skeletal muscle [49, 50]. Moreover, data from Mikhail et al. [32] suggest that people with DM1 exhibit dysregulation of proteins involved in mitochondrial fusion, fission, and mitophagy. This raises the possibility that imbalance in mitochondrial dynamics may contribute to impaired mitochondrial respiratory function in our participants. Moreover, our data showed that these markers of mitochondrial content significantly increased with strength training in the DM1 group, aligning with the improvement seen after aerobic training [32]. Importantly, the training protocol used in the present study also improved lower limb strength and function [33, 34], which is particularly significant given that DM1 causes muscle weakness and atrophy [6]. Our protocol had a very high adherence rate (≈98%) [34] and only required two sessions per week to improve muscle strength and mitochondrial bioenergetics. Given the high prevalence of apathy in the DM1 population (≈40%) [51], a low training frequency appears as a particularly suitable option for this population.

The lower respiration normalized to OXPHOS protein content seen in DM1 participants is indicative of intrinsic alterations in mitochondrial bioenergetics and suggests that mitochondrial quality is impaired in DM1. This observation is consistent with the hypothesis that disease‐related factors, such as RNA toxicity, impair mitochondrial function [32]. Interestingly, three participants presented a decreased mitochondrial mass assessed with the VDAC1 marker, but higher expression of subunit I and/or IV after training. We could have expected a common point between these three participants, which is not the case. One could speculate that these participants could have potentially developed a different compensatory mechanism following strength training. Otherwise, various disease durations and severities among our participants could have led to pre‐training compensatory mechanisms, such as exhibiting the highest percentages of myofibers expressing high subunit complexes I and IV. In brief, the variability in baseline levels and exercise‐induced adaptations may stem from the heterogeneous pathophysiological features of DM1 [52]. This variability among DM1 participants was also evident at the transcriptomics level. Indeed, a transcriptomic analysis of *vastus lateralis* samples from a male DM1 cohort who underwent a 12‐week strength training program revealed that exercise‐induced transcriptomic changes correlated with clinical outcomes only at the individual level [49]. These findings suggest that individual‐level transcriptomic analyses could offer valuable insight in our cohort of women [49].

Contrary to previous reports of elevated ROS levels in DM1 [24, 53, 54], our results showed that DM1 participants had significantly lower H_2_O_2_ emission at baseline compared to unaffected individuals. The lower absolute H_2_O_2_ emission observed in DM1 participants may suggest alterations in ROS buffering capacity, although further measurements of antioxidant defenses are needed to confirm this. Interestingly, after 12 weeks of strength training, mitochondrial H_2_O_2_ emission normalized to mitochondrial content was reduced in DM1 participants, suggesting a protective effect of exercise on mitochondrial ROS production. These results are strengthened by the observed decrease in free radical leak, indicating that 12 weeks of strength training led to a reduction in the rate of electrons leaking from the electron transport chain at a given rate of oxygen consumption. To our knowledge, no study has evaluated mitochondrial ROS production in DM1 participants following exercise training. However, García and colleagues found that fibroblasts isolated from individuals with DM1 displayed elevated H_2_O_2_ levels, an effect that was reversed by metformin administration [24]. Metformin, like exercise, activates the AMPK pathway, which has positive impacts on mitochondria and neuromuscular disorders [55, 56]. Interventions that target AMPK, such as exercise and Metformin, appear beneficial to DM1 patients [24, 56]. It is worth mentioning that DM1 is a disease with high variability in its clinical presentation. With an average of 431 CTG repeats and the ability to attend and participate in a strength training program, our participants were in relatively good physical condition compared to what could be reported for the DM1 population where CTG repeat expansion reaching more than 1000 is observed in more severe phenotypes. In the study of Mikhail et al., where they conducted an aerobic training intervention, the mean CTG repeats was 645 [32]. This could potentially explain why our participants did not exhibit higher ROS levels compared to unaffected individuals.

We also assessed histological markers of skeletal muscle integrity and denervation. Our analysis of the NCAM+ fibers, a widely used marker of muscle denervation [39, 40], showed that individuals with DM1 tend to have more NCAM+ fibers compared to healthy individuals. Our results are in line with recent data highlighting neuromuscular junction abnormalities [16]. To expand on the present findings, future research should include analysis of neuromuscular junctions in skeletal muscle tissue [57] and/or assess mRNA expression profiles of AChR subunits and denervation‐associated markers before/after exercise. Following strength training, even though there was no significant difference between DM1‐PRE and DM1‐POST, the trend vanished between DM1‐POST and unaffected groups. The potential protective effect of strength training on myofiber innervation aligns with a study showing reduced NCAM1 transcript levels after a 5‐month strength training in overweight older adults [58]. Additionally, we observed a higher proportion of centrally located nuclei and nuclear clumps in DM1 participants, which are clinical features exhibited in muscle biopsies from individuals with DM1 [3, 59]. Interestingly, we observed nuclear clumps in DM1 participants at various stages of the disease (i.e., juvenile, late‐onset), supporting previous findings conducted on end‐stage individuals only [60]. Surprisingly, we saw ≈10% of fibers with damaged laminin (i.e., inward folding or rupture of the laminin membrane) in DM1 participants at baseline. To our knowledge, no other study has reported laminin damage in individuals with DM1. While some histological abnormalities persisted post‐intervention, percentages of fibers with centrally located nuclei, damaged laminin and nuclear clumps were reduced by the 12‐week strength training. In previous articles, we reported that this intervention led to significant improvements in muscle fiber size [33, 34], reinforcing its beneficial role in muscle integrity.

### 4.1 Limitations of the Study

This study is limited by its small sample size and tissue availability. Despite these limitations, it is important to note that conducting a structured strength training regimen with multiple biopsies in a population highly affected by a rare disease characterized by low motivation is a considerable achievement. Future studies should include body composition measures to assess physiological improvements beyond histological findings. The integration of electron microscopy, along with analyses of DMPK protein levels and mitochondrial dynamics markers, could have provided more comprehensive insights into the underlying mechanisms of DM1.

## 5 Conclusion

Compared to age‐matched unaffected participants, women with DM1 exhibited reduced mitochondrial respiration and altered H_2_O_2_ emission, along with diminished muscle integrity. A 12‐week strength training program, in addition to the clinical gains [34], significantly improved mitochondrial respiration and content, diminished H_2_O_2_ emission and enhanced muscle integrity. These molecular and histological benefits highlight strength training as a promising strategy for improving muscle physiology and, therefore, overall health in women with DM1.

## Author Contributions

Conception and design of the study: E.D., G.G., J.‐P.L.‐G., and A.V. Data acquisition and analysis: E.D., G.H.‐B., A.V., C.L., G.G., J.A.M., J.‐P.L.‐G., L.G.‐C., M.D., M.‐P.R., O.C., A.A., V.D.L., and V.M. Interpretation of the data: E.D., G.H.‐B., A.V., C.L., G.G., J.A.M., J.‐P.L.‐G., L.G.‐C., M.D., M.‐P.R., O.C., V.D.L., and V.M. Draft of the manuscript: E.D., A.E., L.G.‐C., O.C., A.A., V.D.L., J.‐P.L.‐G., and V.M. All authors revised the manuscript and approved its final version. All authors agree to be accountable for all aspects of the work in ensuring that questions related to the accuracy or integrity of any part of the work are appropriately investigated and resolved. All persons designated as authors qualify for authorship, and all those who qualify for authorship are listed.

## Conflicts of Interest

E.D. has consulted or currently consults for Dyne Therapeutics, PepGen, Avidity, AMO Pharma and Vertex Pharmaceuticals, and has received research funding from Vertex Pharmaceuticals. The funders had no role in the design of the study; in the collection, analyses or interpretation of data; in the writing of the manuscript; or in the decision to publish the results. The other authors declare no conflicts of interest.

## Supporting information


**Table S1:** List of antibodies used for western blotting and histological staining.

## Data Availability

The data that support the findings of this study are available on request from the corresponding author. The data are not publicly available due to privacy or ethical restrictions.
