# Supplementary material for: A 12‐Week Strength Training Improves Mitochondrial Respiration, H2O2 Emission and Skeletal Muscle Integrity in Women With Myotonic Dystrophy Type 1
Source: Acta Physiol (Oxf). 2025 Nov 16;241(12):e70135. doi: 10.1111/apha.70135 (PMC12619986; doi:10.1111/apha.70135)
Supplement: Supplementary file 1 — Table S1: List of antibodies used for western blotting and histological staining. [file APHA-241-e70135-s001.docx]

**Supplementary Material**

Table S1: List of antibodies used for western blotting and histological staining

| Antibody | Source/product no. | Dilution |
| --- | --- | --- |
| NDUFB8 (mouse IgG1) | Abcam #ab110242 | 1/100 |
| MTCO1 (mouse IgG2a) | Abcam #ab 14705 | 1/100 |
| VDAC1 (mouse IgG2b) | Abcam #ab14734 | 1/100 |
| Laminin (rabbit) | MiliporeSigma #L9393 | 1/750 |
| OXPHOS Cocktail (rodent) | Abcam #ab110413 | 1/250 |
| NCAM (mouse IgG1) | BD Science #347740 | 1/50 |
| Dystrophin (mouse IgG2b) | Milipore Sigma #D8168 | 1/500 |
